# Clinical Outcomes Related to the Use of Bendamustine Therapy for Multiple Myeloma Patients Relapsed/Refractory to Immunomodulatory Drugs and Proteasome Inhibitors

**DOI:** 10.4274/tjh.2016.0397

**Published:** 2017-08-02

**Authors:** Fevzi Fırat Yalnız, Nihan Akkoç, Ayşe Salihoğlu, M. Cem Ar, Şeniz Öngören, A. Emre Eşkazan, Teoman Soysal, Yıldız Aydın

**Affiliations:** 1 İstanbul University Cerrahpaşa Faculty of Medicine, Department of Internal Medicine, Division of Hematology, İstanbul, Turkey; 2 İstanbul University Cerrahpaşa Faculty of Medicine, Department of Internal Medicine, İstanbul, Turkey

**Keywords:** Multiple myeloma, Relapse refractory, Bendamustine

## Abstract

**Objective:**

Multiple myeloma patients who are relapsed or refractory to both proteasome inhibitors (PIs) and immunomodulatory drugs (IMiDs) have been reported to have poor outcomes. Bendamustine has been reported to have an antitumor effect in newly diagnosed as well as relapsed/refractory multiple myeloma (RRMM). The aim of this retrospective study was to evaluate the efficacy of bendamustine therapy in heavily pretreated MM patients who were refractory to PIs and IMiDs.

**Materials and Methods:**

Nineteen RRMM patients treated either with bendamustine and steroids (n=13) or a combination of bendamustine with novel drugs (n=6) were included. The median number of previous treatment lines was 5 (minimum-maximum: 3-8) and median time from diagnosis was 6 years (minimum-maximum: 1-16). All of the patients were resistant to at least one of the IMiDs and one of the PIs. Bendamustine was given at doses ranging from 90 mg/m^2^ to 120 mg/m^2^ on days 1 and 2 of 28-day cycles.

**Results:**

A median of 2 (minimum-maximum: 1-8) treatment cycles was administered per patient. The toxicity of bendamustine was mild and mostly of hematological origin. No complete remission was achieved. There was partial remission and stable disease in 21% and 11% of the patients, respectively. Sixty-eight percent of patients had progressive disease. The median progression-free survival and overall survival was 2 and 4 months, respectively.

**Conclusion:**

Bendamustine therapy was well tolerated but showed limited anti-myeloma activity in heavily pretreated patients who were refractory to IMiDs and PIs.

## Abstract (tr)

**Amaç:**

Proteazom inhibitörleri (PIs) ve immünomodülatuvar ilaçlar (IMiDs) içeren tedavi rejimlerine relaps refrakter multipl miyelom (RRMM) hastalarının prognozu oldukça kötüdür. Bendamustin yeni tanı almış ve RRMM hastalarında etkinliği bildirilmiş bir ajandır. Bu retrospektif çalışmanın amacı, öncesinde yoğun tedavi almış, PIs ve IMiDs tedavilerine RRMM hastalarında bendamustin tedavi etkinliğinin ortaya konmasıdır.

**Gereç ve Yöntemler:**

On dokuz RRMM hastasına bendamustin steroid (n=13) veya diğer ajanlarla kombine (n=6) edilerek verildi. Hastaların bendamustin tedavisi öncesi almış oldukları ortanca tedavi sayısı 5 (minimum-maksimum: 3-8), MM tanısından itibaren geçen zaman ortanca 6 yıl (minimum-maksimum: 1-16) olarak tespit edildi. Çalışmaya dahil edilmiş tüm hastalar en az bir IMiDs ve bir PIs dirençli idi. Bendamustin 90 mg/m^2^-120 mg/m^2^ dozlarında 28 günlük tedavi sikluslarının 1. ve 2. günlerinde verildi.

**Bulgular:**

Hastalar ortanca 2 (minimum-maksimum: 1-8) siklüs tedavi aldı. Bendamustin kaynaklı toksisite hafif ve genel olarak hematolojik orjinli tespit edildi. Hiçbir hastada tam remisyona elde edilemedi. Hastaların %21 ve %11’inde sırası ile kısmi remisyon ve stabil hastalık safhasına ulaşıldı. Hastaların %68’inde hastalık progresyonu saptandı. Ortanca progresyonsuz sağkalım ve genel sağkalım sırası ile 2 ve 4 ay olarak tespit edildi.

**Sonuç:**

IMiDs ve PIs dirençli hastalarda bendamustin tedavisi iyi tolere edilmesine rağmen kısıtlı anti-miyelom aktivitesi göstermiştir.

## INTRODUCTION

Multiple myeloma (MM) is the second most common hematological malignancy, accounting for an estimated 1% of all cancers [1]. Introduction of high-dose chemotherapy followed by stem cell rescue and novel treatment modalities such as immunomodulatory drug (IMiD) agents and proteasome inhibitors (PIs) over the past 20 years have led to improved survival rates in patients with MM [2,3]. Recently, the United States Food and Drug Administration approved two monoclonal antibodies indicated for the treatment of MM, which will further help improve the response and survival rates in relapsed refractory multiple myeloma (RRMM). Despite advances in its treatment, MM is still considered to be an incurable disease. For patients who relapse after treatment with novel agents therapeutic strategies are inadequate and usually result in a dismal prognosis. While some salvage treatments exist, patients may not respond to them or may be unable to tolerate them due to toxicities.

Bendamustine is a nitrogen mustard-based alkylating agent shown to be effective in the treatment of various hematologic malignancies. It can be safely administered to patients both with mild to moderate renal insufficiency and moderate hepatic insufficiency [4,5].

Bendamustine has been used for more than a decade for the treatment of MM, either as the sole therapy or in combination with steroids and other chemotherapeutics including novel agents [6]. Considerable efficacy has been reported in newly diagnosed as well as RR patients [7,8].

In this retrospective analysis we tried to explore the real-life effectiveness and safety of bendamustine in heavily pretreated MM patients refractory to IMiDs and PIs.

## MATERIALS AND METHODS

Patients were identified by reviewing the medical records at the Hematology Department of Cerrahpaşa Medical Faculty, İstanbul University. This retrospective study included 19 patients who were RR to at least one of the IMiDs (thalidomide and lenalidomide) and one of the PIs (carfilzomib and bortezomib). Patient characteristics before bendamustine treatment are shown in Table 1.

Bendamustine was given either with steroids (n=13) or in combination with novel agents (n=6) between January 2012 and May 2015 (Table 1). Bendamustine dosage varied from 90 mg/m^2^ to 120 mg/m^2^ and it was administered intravenously on days 1 and 2 of a 28-day cycle as per the protocol described in previous studies [9,10,11]. Bendamustine was combined with lenalidomide and dexamethasone in three patients and with thalidomide and bortezomib in one patient each, respectively. Dexamethasone was given at up to 160 mg per cycle as tolerated. Patients received cotrimoxazole, acyclovir, and fluconazole prophylaxis during treatment.

Treatment response was assessed according to the International Myeloma Working Group Consensus Statement for the management, treatment, and supportive care of patients with myeloma [7]. Overall response rate (ORR) was defined to include complete response, very good partial response, partial response (PR), and minimal response. Overall survival (OS) was calculated as the time from the first day of the bendamustine cycle to death or last patient contact. Progression-free survival (PFS) was defined as the time from bendamustine administration to the date at which criteria for progression were met or death, whichever occurred first. Adverse events were recorded and categorized based on the Common Technology Criteria for Adverse Events Version 4.0 (CTCAE). Time-to-event analysis was performed using the Kaplan-Meier method (JMP v Pro 12).

## RESULTS

Nineteen RRMM patients were included in the study. The median age was 62 years (minimum-maximum: 38-83) and there were 12 males (63%). Patients were heavily pretreated with a median number of 5 (minimum-maximum: 3-8) previous lines of therapy. The median time from diagnosis was 6 years (minimum-maximum: 1-16). All included patients had progressed under their last treatment regimen and had been exposed to all effective drugs available in the country prior to treatment with bendamustine.

Patients were not given a fixed number of bendamustine cycles. Treatment was discontinued in the case of considerable toxicity or ineffectiveness (disease progression).

**Figure 1:**
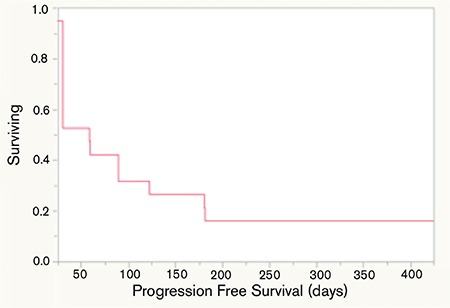
Progression-free survival.

**Figure 2:**
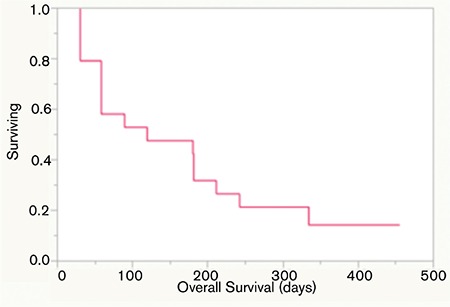
Overall survival.

Following a median of 2 (minimum-maximum: 1-8) treatment cycles, 4 patients showed PR (21%) and 2 patients had stable disease (11%), while in the rest of the patients the disease progressed (68%) (Table 1). Median PFS was 59 days (minimum-maximum: 14-425) (Figure 1) and OS was 120 days (minimum-maximum: 31-456) (Figure 2). Eight patients died during the first 2 months of treatment due to disease progression.

**Figure 3:**
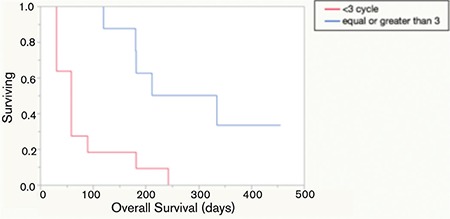
Overall survival based on treatment cycles.

Only eight of the patients were able to receive 3 or more cycles of bendamustine while in the rest of the cases treatment had to be discontinued due to disease progression. Median OS for patients treated with ≥3 and <3 cycles of bendamustine was 274 and 59 days, respectively (Figure 3).

Bendamustine was well tolerated in patients who received it combined with steroids or with novel agents (IMiDs and PIs). The most commonly observed grade 3-4 adverse events included mild to moderate hematological toxicities. Among them, 12 (55%) patients had neutropenia, 5 (23%) patients had thrombocytopenia, and 2 (9%) patients had anemia. Apart from hematological toxicities, 2 (10%) patients developed lower respiratory tract infections of bacterial origin (CTCAE grades 3 and 4). Those patients were hospitalized and treated successfully with intravenous antibiotics. Treatment-related CTCAE grade 3-4 toxicities are summarized in Table 2.

## DISCUSSION

MM patients who are RR to treatment with IMiDs and bortezomib have been reported to have poor outcomes. According to a recent International Myeloma Working Group study, the median OS and PFS of patients refractory to IMiDs and bortezomib were found to be 9 and 5 months, respectively [12]. Options are very limited for those who become resistant to these agents and the vast majority of these patients are unable to tolerate most regimens due to toxicities. Bendamustine could be an option for these patients because of its low toxicity profile. Several clinical studies have demonstrated the effectiveness of bendamustine combined with novel agents in the first-line therapy of MM [13,14,15]. However, published data on bendamustine as monotherapy or in combination with steroids in the treatment of RRMM are limited. Michael et al. [10] in their retrospective analysis looked at the outcomes of RRMM patients (n=39) who were treated with bendamustine as a sole therapy or in combination with steroids. They reported an ORR of 36%, with median event-free survival (EFS) and OS of 7 and 17 months, respectively. In another retrospective study Damaj et al. [11] found an ORR of 30%. Median PFS and OS for the entire cohort were 9.3 and 12.4 months, respectively. An ORR of 59% was reported by Stöhr et al. [16] in heavily treated RRMM patients with a median OS of 17 months and an EFS of 7 months. Recently, Musto et al. [17] published results on 78 MM patients, most of whom were refractory to IMiDs and bortezomib. The ORR was 29%.

We present here a retrospective analysis of patients with RRMM who had been exposed to and were RR to PIs and IMiDs. Unlike our study, in former studies, not all patients had been previously exposed and were refractory to IMiDs and PIs. All of our patients were refractory to their last therapy and all of the patients had been heavily pretreated with all available agents. Furthermore, 5 of them were double-PI and double-IMiD refractory. Bendamustine was considered as a final option for our patients. A median of 2 (minimum-maximum: 1-8) treatment cycles were administered per patient.

The therapy was well tolerated and the most common side effect was neutropenia (Table 2). Bendamustine generally has a favorable toxicity profile with moderate hematological events. Although it has been used for more than a decade for the treatment of myeloma, only a small number of studies reporting its efficacy and safety in different settings and combinations have emerged. A phase II trial defined a dose of 90 mg/m^2^ on days 1 and 4 as the maximum tolerated dose of bendamustine when used in combination with bortezomib [18]. In another study, the maximum tolerated dose was not reached with 75 mg/m^2^ on days 1 and 2 when combined with lenalidomide at 25 mg on days 1 to 21 [19]. In our study, 6 patients were given bendamustine in combination with novel agents (Table 1). Bendamustine at 90 mg/m^2^ on days 1 and 2 of a 28-day cycle was the preferred treatment protocol when administered in combination with the aforementioned novel agents. Twelve of our patients had grade 3-4 neutropenia. Although the efficacy achieved with combinations of bendamustine and other agents is promising, the overlapping myelosuppressive effects of these agents may be problematic. However, there are no clear dosage adjustment recommendations available and due to our small patient size we cannot present a firm conclusion in this regard.

Response rates in our cohort of patients were not as high as was reported in earlier studies. However, patients who could receive 3 or more cycles of bendamustine showed an OS advantage over the patients who were given less than 3 cycles. Nevertheless, statistical comparisons could not be performed due to the small patient numbers. Results of patients with ≥3 cycles were comparable to the best supportive care results in the literature (Figure 3).

## CONCLUSION

Small sample size and the retrospective nature of the study were the two main limitations of our study. Furthermore, cytogenetic profile data of most of the subjects were not available, which is an important issue when evaluating refractoriness to treatment. We think that the main contribution of our study to the current literature is showing the efficacy of bendamustine in heavily pretreated MM patients who were refractory to both IMiDs and PIs. In conclusion, previous studies have shown the efficacy of bendamustine treatment either as monotherapy or combined with novel agents in newly diagnosed MM patients. In RR settings, novel agent-naive patients were also shown to be responsive to bendamustine therapy [16,17]. However, we did not observe a benefit of bendamustine treatment in patients who were refractory to IMiDs and PIs. It is important to reiterate that our sample size does not permit us to make a precise statement. However, based on our experience with this relatively small number of patients, there is no clear recommendation to be made for the use of bendamustine in IMiD- and PI-resistant heavily pretreated MM patients. Such patients should be encouraged to participate in clinical trials evaluating new approaches.

## Figures and Tables

**Table 1:**
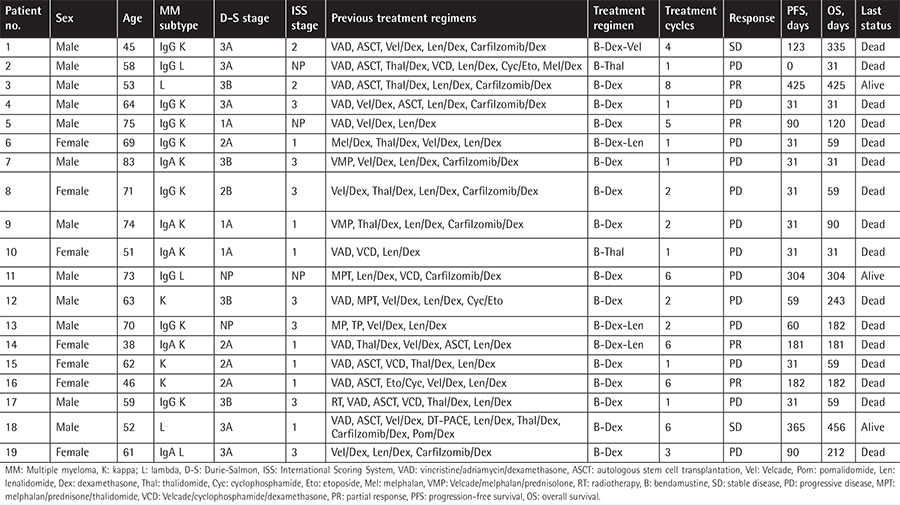
Baseline characteristics and bendamustine treatment outcomes.

**Table 2:**
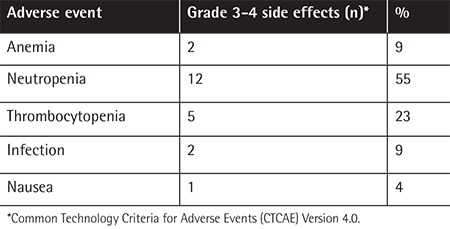
Treatment-related adverse events.
